# Underwater Distortion Target Recognition Network (UDTRNet) via Enhanced Image Features

**DOI:** 10.1155/2021/4193625

**Published:** 2021-10-22

**Authors:** Lei Cai, Chuang Chen, Haojie Chai

**Affiliations:** ^1^School of Artificial Intelligence, Henan Institute of Science and Technology, Xinxiang 453003, China; ^2^School of Information Engineering, Henan Institute of Science and Technology, Xinxiang 453003, China

## Abstract

It is difficult for the autonomous underwater vehicle (AUV) to recognize targets similar to the environment in lacking data labels. Moreover, the complex underwater environment and the refraction of light cause the AUV to be unable to extract the complete significant features of the target. In response to the above problems, this paper proposes an underwater distortion target recognition network (UDTRNet) that can enhance image features. Firstly, this paper extracts the significant features of the image by minimizing the info noise contrastive estimation (InfoNCE) loss. Secondly, this paper constructs the dynamic correlation matrix to capture the spatial semantic relationship of the target and uses the matrix to extract spatial semantic features. Finally, this paper fuses the significant features and spatial semantic features of the target and trains the target recognition model through cross-entropy loss. The experimental results show that the mean average precision (mAP) of the algorithm in this paper increases by 1.52% in recognizing underwater blurred images.

## 1. Introduction

Underwater target recognition has difficulties in sample data collection and labeling, making it difficult to obtain labeled sample datasets. Unsupervised representational learning can extract significant features of images from unlabeled datasets and use them for target classification and detection tasks. This method can improve the accuracy of underwater target recognition effectively in the case of insufficient tags. Moreover, unsupervised representational learning ignores some details of the image and only learns distinguishable features, which can also improve the recognition speed of the algorithm.

However, the scattering and refraction of light in the underwater environment cause the target to be blurred and distorted in the images taken by the AUV. Shoals of fish, currents, and complex underwater terrain can obscure the target. In this case, unsupervised representational learning is unable to extract the complete significant features for target recognition. The graph structure establishes the topology of correlation between nodes through vertices and edges and contains rich spatial semantic information. Semantic relationship graphs can compensate for incomplete significant features.

Graph convolutional networks (GCNs) can extract features of graph structures and gain the spatial semantic relations of targets effectively. However, the spatial semantic relationship graphs of the targets are usually static graphs obtained by computing the label co-occurrence relationships in the whole dataset. In the underwater environment, the number of sample data from different classes in the dataset is unevenly distributed. In this case, a static spatial semantic relationship graph will reduce the generality of the model [1]. Constructing a dynamic spatial semantic relationship graph can improve the robustness of the algorithm.

To address the above problems, this paper proposes an underwater distortion target recognition network (UDTRNet) via enhanced image features. The method allows fast recognition of underwater distortion targets in the absence of significant features.

The following are the main contributions in the methodology of this paper:

1. In this paper, the original sample data are compared with positive and negative samples in the feature space, respectively. The proposed algorithm trains the feature extraction network by minimizing the InfoNCE loss function to extract the visual significant features of the target. This method can improve the target recognition accuracy in the absence of data labels.
2. This paper adds the label information of the current image in the static correlation matrix. The proposed algorithm constructs the dynamic correlation matrix to represent the spatial semantic relationships of the targets. This matrix can extract dynamic spatial semantic features to compensate for the lack of significant features caused by target distortion and occlusion.
3. The proposed algorithm fuses the significant features and spatial semantic features of the target and trains the target recognition model through cross-entropy loss. The experimental results show that the method effectively solves the problem of low recognition accuracy when the target is distorted and obscured.

The rest of this paper is presented as follows.  Section 2 describes the related work.  Section 3 introduces the visual significant feature extraction model, the spatial semantic feature extraction model, and the underwater distortion target recognition algorithm via enhanced image features.  Section 4 verifies the effectiveness of the methods in this paper through simulation experiments.  Section 5 concludes the paper.

## 2. Related Work

Absorption and scattering of light cause difficulties in underwater image acquisition. It is expensive to produce large annotated underwater datasets. In the absence of data tags, AUV has difficulty in identifying targets that are similar to their environment. Unsupervised representational learning can extract distinguishable features of images using unlabeled data. Wang et al. [2] proposed an adversarial correlated autoencoder (AdvCAE) for unsupervised multiview representation learning. This method eliminates the differences in data from multiple views due to different distributions. Also, Han et al. [3] proposed a semisupervised multiview manifold discriminant intact space (SM2DIS) learning method for image classification. This method learns the complete feature representation by multiview data. Le-Khac et al. [4] summarized the existing literature on contrast learning and proposed a generalized framework for contrast representation learning. The framework simplifies and unifies many different contrast learning algorithms and addresses the application of the contrast learning framework to the field of computer vision. Chen et al. [5] extended the existing contrast learning algorithm by embedding an attention mechanism and proposed an attention-augmented contrastive (A2C) learning method. The method can improve the learning efficiency and generalization ability of the algorithm. Li et al. [6] proposed an intermediate-level feature representation framework for unsupervised representation learning via sparse autoencoders. Experimental results show that the method reduces the number of parameters for unsupervised representation learning.

The complex underwater environment and light refraction make it difficult for the AUV to extract the complete significant features of the target. The images contain rich spatial semantic relationships. Su et al. [7] proposed a new multigraph embedding discriminative correlation feature learning algorithm. The method captures the intrinsic geometric structure of each view and learns nonlinear correlation features with good recognition ability. Ma et al. [8] proposed a multiscale spatial context-based deep network for semantic edge detection (MSC-SED). The network obtains rich multiscale features while enhancing high-level feature details. Yang et al. [9] proposed to combine structured semantic relevance to solve the problem of missing labels in multilabel learning. Zhao et al. [10] designed a multitasking framework to jointly handle the weather clues to the segmentation task and the weather classification task. This method solves the problem of poor performance of single weather tag classification. Khan et al. [11] proposed a new multilabeled deep GCN. The network can extract discriminative features from the irregular structure to enhance the classification results. Nauata et al. [12] proposed to model the complex relationships between labels through a structured inference neural network. The experimental results show that the method improves the applicability and robustness of the algorithm. Chen and Gupta [13] proposed a spatial memory network (SMN) that can model instance-level contexts. This method can improve the target detection accuracy by using the contextual relationship of the object.

For the problems of underwater environment interference and algorithm real time, Cai et al. [14] proposed a collaborative multi-AUV target recognition method based on migration reinforcement learning. Zhang et al. [15] proposed a semantic spatial fusion network (SSFNet) to bridge the gap between low-level and high-level features. Moniruzzaman et al. [16] proposed a Faster R-CNN algorithm using the Inception V2 network. This method can improve the average detection accuracy of the algorithm in the case of a small difference between the target and the surrounding boundary. Wang et al. [17] proposed a multiview visual-semantic representation method for few-labeled visual recognition (MV^2^S). The method uses the visual and semantic representation of the image to predict the class of the image. To improve the convergence speed of the algorithm, Cai et al. [18] designed an effective outer space acceleration algorithm. Sun and Cai [19] proposed a multi-AUV target recognition method based on GAN-meta learning. The experiment result shows that this method can improve the generalization ability of the model. Cai et al. [20] proposed a maneuvering target recognition method based on multiview optical field reconstruction. This method can ignore the effect of shooting angle on target recognition results. Chen et al. [21] proposed a new iterative visual inference framework. The framework effectively improves the target recognition accuracy. To solve the problem of data double-computation, Cai et al. [22] proposed a multiview optical field reconstruction method based on migration reinforcement learning.

## 3. Proposed Method

**Figure 1:**
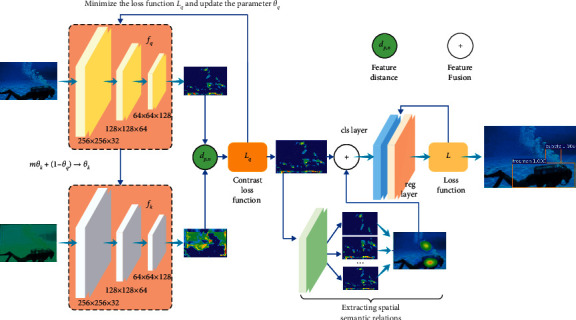
Underwater distortion target recognition network.

This paper proposes the UDTRNet that can enhance image features. This method can make up for the lack of visual significant features through the spatial semantic features of the target. Firstly, the proposed algorithm trains the feature extraction network through the InfoNCE loss function to extract the visual significant features of the target. Then, the dynamic correlation matrix is constructed to represent the spatial semantic relationship of the target, and the spatial semantic features of the target are extracted through this matrix. Finally, this paper fuses the significant features and spatial semantic features of the target and trains the target recognition model through cross-entropy loss. The algorithm effectively solves the problem of low accuracy of target recognition under interference such as distortion and occlusion. The overall process of the algorithm is shown in Figure 1.

### 3.1. Visual Significant Feature Extraction Model

**Figure 2:**
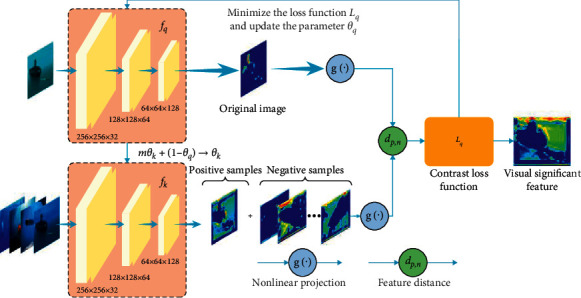
Visual significant feature extraction model.

Unsupervised representation learning can ignore some details of the image. This paper trains a significant feature extraction model to extract distinguishable feature representations of images. The training process is shown in Figure 2.

This paper uses ResNet as the network structure of the significant feature extraction model *f*(·). The last fully connected layer of the network outputs a 128-dimensional feature vector. The representation *h* of the image is obtained by normalizing the feature vector, which is expressed as *h*=*f*(*X*). Then, the characterization vector of the image is nonlinearly projected into the vector *z* through the fully connected layer *g*(·). This method can amplify invariant features and enhance the ability of the network to recognize targets in different views. This paper trains the coding network *f*(·) by minimizing the loss function.

In this paper, *N* original images are randomly enhanced. The images in the real underwater scene have the characteristics of blurring, distortion, and incompleteness. This paper uses random cropping, random color distortion, and random Gaussian blur to obtain enhanced samples of the original image. The number of negative samples is the important factor affecting model representation learning. This paper constructs the feature library to store all the enhanced samples in the training process. For the images input by the feature extraction network, there are positive samples *k*^+^ from the same image as the input samples and negative samples *k*^−^ from different images in the feature library.

The significant feature extraction network maximizes the consistency among different enhanced views of the same image and minimizes the consistency among enhanced views of different images. This method can learn the characterization of image distinguishability. This paper designs a loss function so that the representation of the input image is similar to the positive samples and not similar to the negative samples. The similarity of the images is expressed by the cosine similarity of the feature vectors, which is calculated as follows:

(1) $$\begin{array}{c} \text{sim}\left(z_{q},z_{k}\right)=\frac{z_{q}\cdot z_{k}}{\left\|z_{q}\right\|\left\|z_{k}\right\|}, \end{array}$$

where *z*_*q*_=*g*(*h*_*q*_) denotes the nonlinear projection of the representation vector of the input image and *z*_*k*_ denotes the nonlinear projection of positive or negative sample representations in the feature library. The loss function of the significant feature extraction model is given by

(2) $$\begin{array}{c} {ℒ}_{q}=-\log\frac{\exp\left(\text{sim}\left(z_{q},z_{k^{+}}\right)/\tau \right)}{\exp\left(\text{sim}\left(z_{q},z_{k^{+}}\right)/\tau \right)+\sum_{k^{-}}\exp\left(\text{sim}\left(z_{q},z_{k^{-}}\right)/\tau \right)}, \end{array}$$

where *q* is the feature representation of the input image, *k*^+^ is the feature representation of positive samples, *k*^−^ is the feature representation of negative samples, and *τ* is used to zoom in on the similarity metric of the image representation.

The feature library can make the number of negative samples larger and improve the training effect. However, the phenomenon also increases the difficulty in updating the feature library encoder *f*_*k*_. This paper dynamically updates the feature library encoder *f*_*k*_ by the encoder *f*_*q*_ of the input samples. The parameters of the encoder *f*_*q*_ and *f*_*k*_ are denoted as *θ*_*q*_ and *θ*_*k*_, respectively. *θ*_*k*_ is updated as follows:

(3) $$\begin{array}{c} m\theta_{k}+\left(1-\theta_{q}\right)⟶\theta_{k}, \end{array}$$

where the momentum coefficient *m* ∈ [0,1). During the training process, *θ*_*q*_ updates the parameters by stochastic gradient descent. When *θ*_*q*_ is updated, *θ*_*k*_ updates the parameters according to the above process. After completing the training, the encoder *f*_*q*_ can extract the significant features of the image. The significant features of the images are as follows:

(4) $$\begin{array}{c} f=f_{q}\left(x\right), \end{array}$$

where *x* is the input test image and *f*_*q*_ is the encoder with completed training.

### 3.2. Spatial Semantic Feature Extraction Model

**Figure 3:**
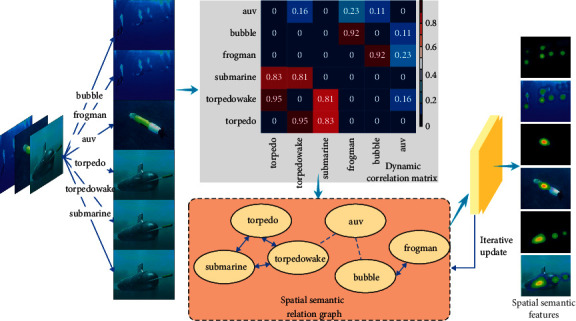
Spatial semantic feature extraction model.

The target in the underwater image is distorted. The algorithm is unable to extract the significant features of the target completeness. This phenomenon can reduce the accuracy of target recognition. This paper extracts the spatial semantic features among nodes by edge traversal and updating the nodes in the graph. The spatial semantic feature extraction model is shown in Figure 3.

This paper constructs the spatial semantic relation graph *G*={*V*, *E*} for the target, where *V* is the set of nodes and *E* is the edge set. The node indicates the category of the target. The edges represent the spatial semantic relationships among different targets. Assume that the dataset includes *C* target categories. The set of nodes *V* can be represented as {*v*_0_, *v*_1_,…, *v*_*C*−1_}. The element *v*_*c*_ indicates the category *c*. The edge set *E* is the correlation matrix that can represent the correlation among different objectives. However, the static correlation matrix mainly explains the co-occurrence of labels in the training dataset. The correlation matrix of each input image is fixed. This matrix does not explicitly utilize the content of each input image. This paper constructs the local correlation matrix *B* for each specific input image. The global correlation matrix and the local correlation matrix are fused as the overall correlation matrix. The results are as follows:

(5) $$\begin{array}{c} A=\omega_{E}E+\omega_{B}B=\left\{\begin{array}{ccc} a_{00} & \cdots & a_{0\left(C-1\right)}\\ \vdots & \ddots & \vdots \\ a_{\left(C-1\right)0} & \cdots & a_{\left(C-1\right)\left(C-1\right)} \end{array}\right\}, \end{array}$$

where *ω*_*E*_ and *ω*_*B*_ denote the weights. The element *a*_*cc*′_ denotes the probability of having both target *c*′ and target *c* in the image, i.e., the correlation between target *c*′ and target *c*. This paper uses the labels of the training set to calculate the correlation between different category pairs in the input images.

The spatial semantic relationship of the target is learned through the spatial semantic relationship diagram. Each node *v*_*c*_ has a correlation *h*_*c*_^*t*^ at time step *t*. This parameter indicates the degree of correlation among the node and other nodes. In this article, each node corresponds to a specific target category. The spatial semantic feature extraction model aims to learn the spatial semantic relationship among objects. When the step length *t*=0, the spatial semantic relationship and the feature vector are initialized and expressed as *h*_*c*_^0^=*f*. The framework aggregates information from neighboring nodes.

(6) $$\begin{array}{c} a_{c}^{t}=\left[\sum_{c^{\prime }}\left(a_{cc^{\prime }}\right)h_{c}^{t-1},\sum_{c^{\prime }}\left(a_{c^{\prime }c}\right)h_{c}^{t-1}\right]. \end{array}$$

The model encourages the dissemination of information among highly correlated nodes. This paper learns spatial semantic relations through information transfer in graphs. The proposed algorithm updates the spatial semantic relations of the target by aggregating the feature vector *a*_*c*_^*t*^. The iterative process is as follows:

(7) $$\begin{array}{c} \left\{\begin{array}{c} z_{c}^{t}=\sigma \left(W^{z}a_{c}^{t}+U^{z}h_{c}^{t-1}\right),\\ r_{c}^{t}=\sigma \left(W^{r}a_{c}^{t}+U^{r}h_{c}^{t-1}\right),\\ \tilde{h_{c}^{t}}=\tanh\left(Wa_{c}^{t}+U\left(r_{c}^{t}⊙h_{c}^{t-1}\right)\right),\\ h_{c}^{t}=\left(1-z_{c}^{t}\right)⊙h_{c}^{t-1}+z_{c}^{t}⊙h_{c}^{t}, \end{array}\right. \end{array}$$

where *σ*(·) is a logarithmic sigmoid function, tanh(*·*) is a hyperbolic tangent function, and ⊙ denotes the multiplication operator between elements. The target node aggregates the information of surrounding nodes to achieve the interaction between the feature vectors corresponding to different nodes. The iterative process lasts for *T* times. The obtained spatial semantic relation is *H*={*h*_0_^*T*^, *h*_1_^*T*^,…, *h*_*C*−1_^*T*^}.

### 3.3. Underwater Distortion Target Recognition Method via Enhanced Image Features

This section extracts the candidate regions of the target on the visual significant feature map of the image. The proposed algorithm fuses visual significant features and spatial semantic features to accomplish target recognition.

This paper obtains the target anchor boxes by sliding the window on the significant feature *f*. The window size is 3*∗*3. The algorithm predicts multiple target anchor boxes simultaneously in each window. The maximum number of anchor boxes per position is denoted as *k*. Each anchor box maps a low-dimensional feature. The features are input to the classification (cls) layer and the regression (reg) layer. The reg layer outputs the coordinates of the vertices of the *k*-group anchor boxes. The cls layer outputs the label and confidence level of the anchor box. For the feature mapping of *W* × *H*, the proposed method generates *k* × *W* × *H* target anchor boxes.

This paper indicates the prediction accuracy of the model by intersection over union (IoU). The model assigns a binary label to each target candidate frame. Candidate boxes with IoU greater than 0.7 are positive labels. Candidate boxes with IoU less than 0.3 are negative labels. If there is no anchor box with IoU greater than 0.7, the algorithm selects the candidate box with the largest IoU as the positive label. In addition, nonpositive and negative labels and candidate frames that cross the image boundary are of no value to the training for the model. This article deletes it to save calculation time.

This paper considers anchor boxes as nodes in the semantic relationship graph. The proposed method fuses the significant features *f*_*c*_ of nodes and spatial semantic features *h*_*c*_^*t*^ to predict the target types of nodes. The fused features are represented as

(8) $$\begin{array}{c} P_{c}=F_{P}\left(f_{c},h_{c}^{T}\right), \end{array}$$

where *F*_*P*_ is a feature fusion output function. This function maps *f*_*c*_ and *h*_*c*_^*T*^ to the feature vector *P*_*c*_. The feature vector *P*_*c*_ includes the significant features and spatial semantic information of the target. This paper feeds this feature vector into a fully connected classification layer to predict the target category score.

The cls layer of the model is used for object classification and outputs the discrete probability distribution for each anchor box. The cls layer outputs a *C*+1-dimensional array *S*. This array represents the probability that the object belongs to *C* categories and background. The array *S* is usually calculated by the fully connected layer using the SoftMax function.

(9) $$\begin{array}{c} S=\left(s^{0},s^{1},\ldots ,s^{C}\right). \end{array}$$

This paper trains the model by minimizing the loss function. The loss function consists of two components: classification loss and regression loss. The calculation is shown as follows:

(10) $$\begin{array}{c} L=\frac{1}{N_{\text{cls}}}\sum_{i}\sum_{c=1}^{C}s_{i}^{*}\log\left(\sigma \left(s_{i}^{c}\right)\right)+\left(1-s_{i}^{*}\right)\log\left(1-\sigma \left(s_{i}^{c}\right)\right)+\lambda \frac{1}{N_{\text{reg}}}\sum_{i}P_{i}^{*}R\left(T_{i}-T_{i}^{*}\right), \end{array}$$

where *i* denotes the number of anchor boxes. *c* is the target category. *s*_*i*_^*c*^ denotes the predicted probability of the target type in anchor box *i*. *s*_*i*_^*∗*^ is the real label of anchor box *i*. *T*_*i*_ denotes the coordinates of the four vertices of the target anchor box. *T*_*i*_^*∗*^ is the vertex coordinates of the real target region. *R* is the smooth L1 function. *s*_*i*_^*c*^ and *T*_*i*_ are given by the classification and regression layers. *N*_cls_ and *N*_reg_ denote the normalization of the loss function. *N*_cls_ is numerically equal to the minimum batch size for training. *N*_reg_ is equal to the number of target anchor boxes. *λ* is the weight. *σ*(*·*) is the sigmoid function.

## 4. Experimental Results and Analysis

In this experiment, training and testing are performed in TensorFlow. The simulation calculation runs on small server (RTX 2080Ti GPU, 64G of RAM, and Win10 64-bit operating system).

### 4.1. Experimental Dataset

In this paper, the three datasets, Cognitive Autonomous Diving Buddy (CADDY) underwater dataset, Underwater Image Enhancement Benchmark (UIEB), and Underwater Target dataset (UTD) are used for training and testing. The visual salient feature extraction model is trained by 13,000 unlabeled images. In addition, 426 labeled images are used to train and test the spatial semantic feature extraction model and target recognition network. The dataset is divided into training set and test set according to the ratio of 6.5 : 3.5.

### 4.2. Implementation Details

The model is trained by the stochastic gradient descent (SGD) optimizer with a weight decay of 0.0005 and a momentum of 0.9. The training batch is 256 and the initial learning rate is 0.01. The whole training process is iterated 70,000 times, in which the learning rate decays at 56,000 and 63,000 iterations with a decay rate of 0.1.

### 4.3. Experimental Results

The model proposed in this paper fuses visual significant features and spatial semantic features for target recognition. This method can solve the problem of low recognition accuracy under interference such as target distortion and blurring. For underwater images with different disturbances, this section designs three sets of simulation experiments to verify the effectiveness of the proposed algorithm. The algorithm evaluation criteria are mAP and recognition time.

For underwater images with different interferences, three sets of simulation experiments are designed to verify the effectiveness of the proposed algorithm.

#### 4.3.1. Conventional Underwater Image Recognition Results

**Figure 4:**
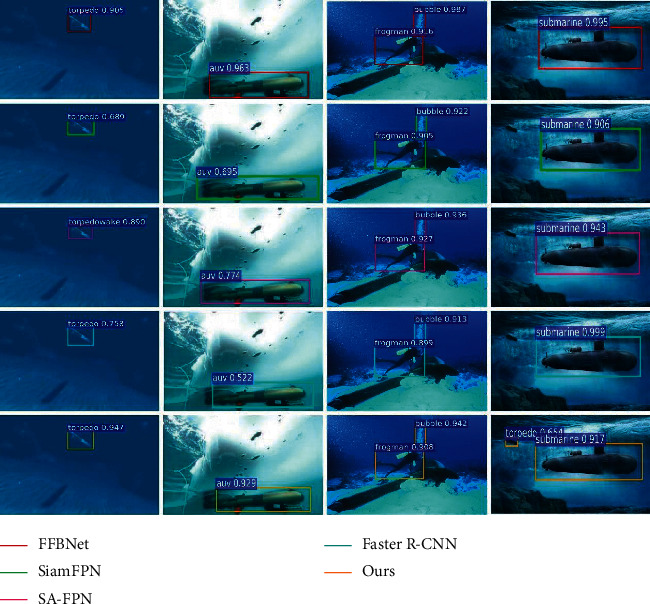
Conventional underwater image target recognition results.

This section evaluates the recognition performance of the proposed algorithm in conventional underwater images and compares it with FFBNet [23], SiamFPN [24], SA-FPN [25], and Faster R-CNN [26]. The recognition results are shown in Figure 4, and the recognition accuracy and recognition speed are shown in Table 1.

As can be seen from Table 1, FFBNet takes 0.09 s to recognize an underwater image, which has the fastest recognition speed among all the compared algorithms. However, the mAP of the algorithm is only 0.7132. On the contrary, Faster R-CNN has the highest mAP and the lowest recognition speed, respectively, 0.7466 and 0.397 s. SiamFPN and SA-FPN greatly reduce the recognition time at the expense of partial accuracy. The proposed algorithm in this paper can better balance the recognition speed and accuracy. In the recognition of conventional underwater images, the overall performance of this proposed algorithm is better than that of FFBNet and Faster R-CNN. At the same time, compared with SiamFPN and SA-FPN, the proposed algorithm has lower recognition time and higher recognition accuracy.

#### 4.3.2. Underwater Blurred Image Recognition Results

**Figure 5:**
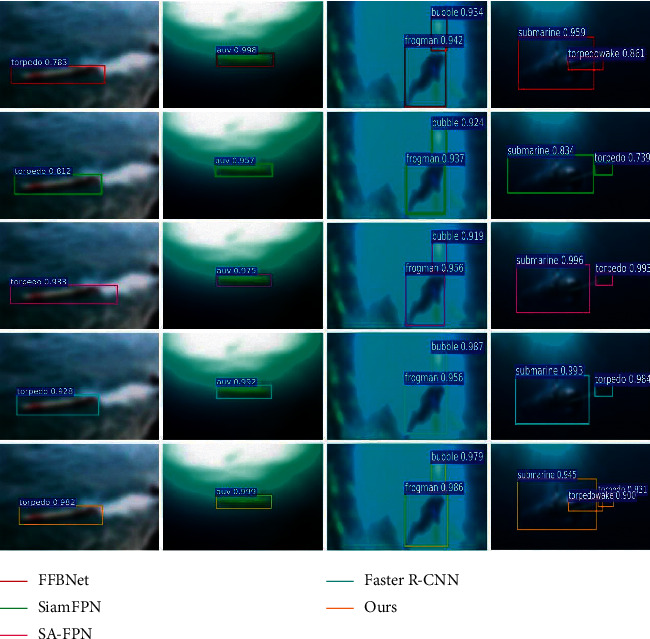
Underwater blurred image recognition results.

This section evaluates the performance of the proposed algorithm to recognize underwater blurred images and compares it with FFBNet [23], SiamFPN [24], SA-FPN [25], and Faster R-CNN [26]. The recognition results are shown in Figure 5. The recognition accuracy and recognition speed of each algorithm are shown in Table 2.

As can be seen in Table 2, the accuracy of each algorithm in recognizing underwater blurred images has decreased. The mAP of the algorithm in this paper is 0.6652, which is ahead of other comparison algorithms. Compared with the state-of-the-art target recognition algorithm SA-FPN, the mAP of the proposed algorithm is improved by 1.52%. Moreover, the algorithm in this paper has a great lead in identifying torpedo, frogman, and submarine targets. The reason is that the method in this paper can enhance the target features through spatial semantic relations.

#### 4.3.3. Underwater Distortion Image Recognition Results

**Figure 6:**
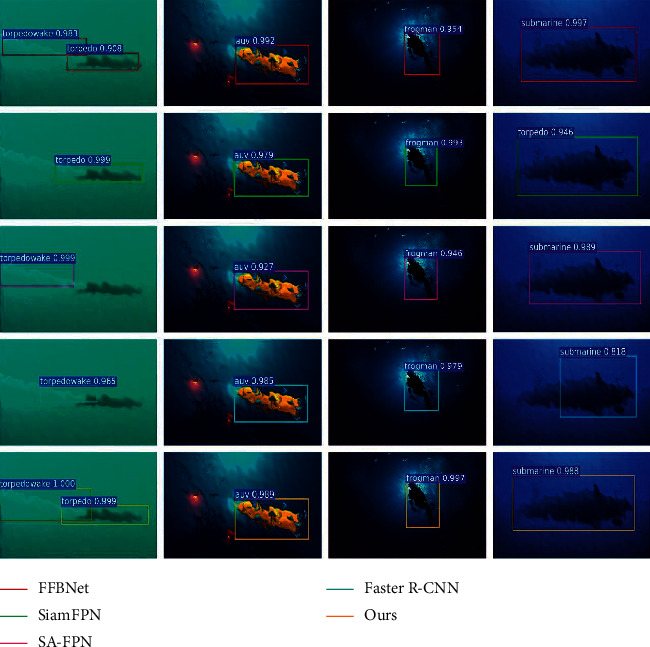
Underwater distorted image recognition results.

This section evaluates the performance of the proposed algorithm to recognize underwater distorted images and compares it with FFBNet [23], SiamFPN [24], SA-FPN [25], and Faster R-CNN [26]. The recognition results are shown in Figure 6. The recognition accuracy and recognition speed of each algorithm are shown in Table 3.

As can be seen from Table 3, the SiamFPN algorithm has the best recognition effect on underwater distorted images. The recognition accuracy is 0.6652, and the recognition speed is 0.225 s. Though the average recognition accuracy of the algorithm in this paper is 1.82% lower than that of SiamFPN, the algorithm has faster recognition speed. This paper also analyzes the recognition results of single-type targets. The algorithm in this paper is more effective in identifying distorted torpedo and frogman targets.

## 5. Conclusion

In the case of many underwater interferences, it is difficult for AUVs to extract the complete significant features of the target. This paper uses spatial semantic features to make up for the lack of distinctive visual features. Firstly, this paper extracts the significant features of the image by minimizing the InfoNCE loss. Secondly, this paper constructs the dynamic correlation matrix to capture the spatial semantic relationship of the target and uses the matrix to extract spatial semantic features. Finally, this paper fuses the salient features and spatial semantic features of the target and then trains the target recognition model through cross-entropy loss. In the recognition of underwater conventional images and distorted images, the comprehensive performance of the algorithm in this paper is better than that of existing algorithms. When recognizing underwater blurred images, the mAP of the algorithm in this paper is improved by 1.52% compared with the existing algorithm.

## Figures and Tables

**Table 1:** Conventional underwater image target recognition accuracy and time.

Method	Torpedo	Torpedo wake	Submarine	Frogman	Bubble	AUV	mAP	Time
FFBNet	0.5397	0.5584	0.8019	0.7775	0.6423	0.9591	0.7132	**0.090**
SiamFPN	0.5855	0.5795	0.8638	0.7444	**0.7012**	**0.9681**	0.7404	0.227
SA-FPN	0.5858	0.7172	0.8733	0.7207	0.6175	0.8312	0.7243	0.240
Faster R-CNN	0.6685	**0.7554**	0.7777	**0.778**	0.6815	0.8182	**0.7466**	0.397
Ours	**0.6851**	0.5682	**0.888**	0.7513	0.6362	0.9478	0.7461	0.215

The bold values indicate excellent indicators of each algorithm.

**Table 2:** Underwater blurred image recognition accuracy and recognition time.

Method	Torpedo	Torpedo wake	Submarine	Frogman	Bubble	AUV	mAP	Time
FFBNet	0.4177	**0.645**	0.6177	0.7097	0.655	0.7695	0.6358	**0.110**
SiamFPN	0.4381	0.4113	0.7005	0.678	0.6798	0.8697	0.6296	0.242
SA-FPN	0.3951	0.5325	0.6749	0.7041	0.6667	**0.9269**	0.6500	0.272
Faster R-CNN	0.4439	0.6091	0.669	0.6669	**0.6828**	0.7156	0.6312	0.397
Ours	**0.5158**	0.579	**0.7633**	**0.7614**	0.5786	0.7932	**0.6652**	0.215

The bold values indicate excellent indicators of each algorithm.

**Table 3:** Underwater distorted image recognition accuracy and recognition time.

Method	Torpedo	Torpedo wake	Submarine	Frogman	Bubble	AUV	mAP	Time
FFBNet	0.465	0.4876	0.7224	0.7222	0.6369	0.8012	0.6392	**0.100**
SiamFPN	0.4909	**0.5808**	0.6815	0.7291	**0.6618**	0.8468	**0.6652**	0.225
SA-FPN	0.4899	0.5462	**0.7734**	0.6734	0.6197	0.833	0.6559	0.239
Faster R-CNN	0.5008	0.5111	0.749	0.6383	0.652	**0.878**	0.6549	0.404
Ours	**0.5296**	0.4873	0.6597	**0.7379**	0.6517	0.8156	0.6470	0.214

The bold values indicate excellent indicators of each algorithm.

## Data Availability

The data used to support the findings of this study are available from the corresponding author upon request.
